# C-Myc/H19/miR-29b axis downregulates nerve/glial (NG)2 expression in glioblastoma multiforme

**DOI:** 10.1016/j.omtn.2024.102120

**Published:** 2024-01-12

**Authors:** Anne S. Boewe, Selina Wrublewsky, Jessica Hoppstädter, Claudia Götz, Alexandra K. Kiemer, Michael D. Menger, Matthias W. Laschke, Emmanuel Ampofo

**Affiliations:** 1 Institute for Clinical and Experimental Surgery, Saarland University, 66421 Homburg, Germany; 2 Department of Pharmacy, Pharmaceutical Biology, Saarland University, 66123 Saarbruecken, Germany; 3 Medical Biochemistry and Molecular Biology, Saarland University, 66421 Homburg, Germany

**Keywords:** MT: Non-coding RNAs, glioblastoma multiforme, NG2, miR-29b, PDGFRα, H19, c-Myc, proliferation, migration

## Abstract

Nerve/glial antigen (NG)2 is highly expressed in glioblastoma multiforme (GBM). However, the underlying mechanisms of its upregulated expression are largely unknown. *In silico* analyses reveal that the tumor-suppressive miR-29b targets NG2. We used GBM-based data from The Cancer Genome Atals databases to analyze the expression pattern of miR-29b and different target genes, including NG2. Moreover, we investigated the regulatory function of miR-29b on NG2 expression and NG2-related signaling pathways. We further studied upstream mechanisms affecting miR-29b-dependent NG2 expression. We found that miR-29b downregulates NG2 expression directly and indirectly via the transcription factor Sp1. Furthermore, we identified the NG2 coreceptor platelet-derived growth factor receptor (PDGFR)α as an additional miR-29b target. As shown by a panel of functional cell assays, a reduced miR-29b-dependent NG2 expression suppresses tumor cell proliferation and migration. Signaling pathway analyses revealed that this is associated with a decreased ERK1/2 activity. In addition, we found that the long noncoding RNA H19 and c-Myc act as upstream repressors of miR-29b in GBM cells, resulting in an increased NG2 expression. These findings indicate that the c-Myc/H19/miR-29b axis crucially regulates NG2 expression in GBM and, thus, represents a target for the development of future GBM therapies.

## Graphical abstract

**Figure:**
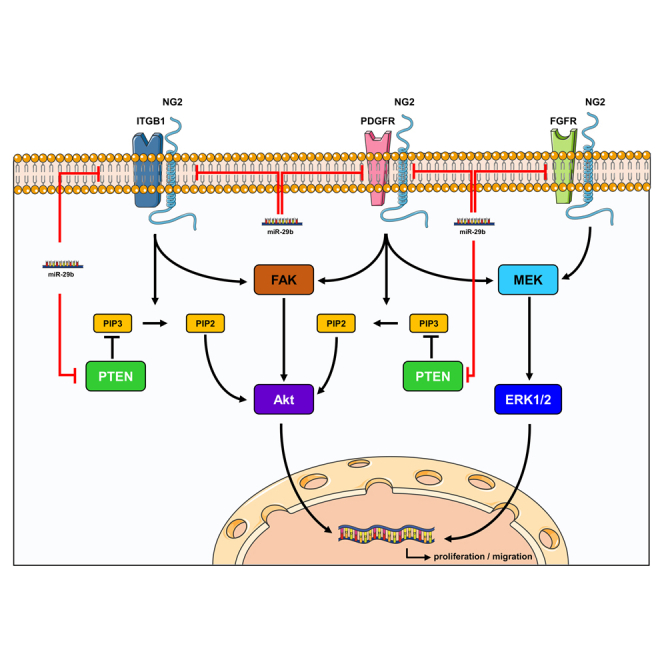

## Teaser

Ampofo and colleagues found that miR-29b reduces NG2 and PDGFRα expression in GBM. Moreover, we identified H19 as a molecular sponge for miR-29b. The expression of H19 is, in turn, induced by the oncogene c-Myc. Taken together, these findings indicate the importance of the c-Myc/H19/miR-29b/NG2 axis for GBM progression.

## Introduction

Glioblastoma multiforme (GBM) with IDH wild type is categorized as grade IV glioma and the most common form of primary brain tumors in adults.^1^ GBM has the highest mortality rate among all brain malignancies, as reflected by a median survival of less than 15 months from the day of diagnosis.^2^^,^^3^^,^^4^ This is due to its highly aggressive invasion of the surrounding tissue as well as its resistance to anticancer drugs and ionizing radiation.^5^

Several studies reported that GBM expresses nerve/glial antigen (NG)2, also known as chondroitin sulfate proteoglycan (CSPG)4, which is a type-1 transmembrane proteoglycan with a core of 290 kDa.^6^ The extracellular domain of the proteoglycan binds to extracellular matrix components, such as collagen type V/VI.^7^ Moreover, NG2 interacts with fibroblast growth factor receptor, integrin β1 (ITGB1) and platelet-derived growth factor (PDGF)α and PDGFRβ, which triggers signaling pathways, including extracellular-signal regulated kinases (ERK)1/2, focal adhesion kinase (FAK) and phosphatidylinositol 3-kinase (PI3K)/Akt.^8^^,^^9^^,^^10^^,^^11^ This, in turn, activates cell proliferation and motility.^12^^,^^13^^,^^14^^,^^15^ Recent findings revealed that one-third of patient-derived GBM cell cultures express NG2 and that these NG2-positive cells proliferate faster and more aggressively *in vivo* than NG2-negative cells.^16^ Therefore, it is not surprising that a high NG2 expression in GBM is associated with a poor patient survival.^17^ However, the mechanisms of this upregulated NG2 expression are still elusive.^18^ So far, only a few transcription factors have been identified that activate or repress the expression of the proteoglycan, such as Sp1.^18^^,^^19^

Besides its transcriptional control, the expression of NG2 is also regulated by microRNAs (miRNAs).^20^ miRNAs are a group of small noncoding RNA fragments that bind to the mRNA and promote its degradation. Many studies reported that miRNAs are implicated in the initiation and progression of GBM, where they exert oncogenic or tumor-suppressive functions.^21^ The microRNA (miR)-29 family (miR-29a, miR-29b, and miR-29c) is downregulated in GBM,^21^^,^^22^ which promotes the proliferation and migration of GBM cells and reduces their sensitivity to temozolomide (TMZ).^23^^,^^24^^,^^25^ Moreover, low serum exosomal miR-29b levels in GBM patients are associated with an unfavorable prognosis.^26^

*In silico* analyses predict that miR-29b targets the 3′UTR of NG2. Therefore, we herein hypothesized that overexpression of miR-29b in GBM counteracts the aggressiveness of NG2-positive GBM. To test this, The Cancer Genome Atlas (TCGA)-based data were used to analyze levels of miR-29b and its target genes in different brain cancer entities. Moreover, we investigated the effect of miR-29b on NG2-mediated cellular functions. Furthermore, the effect of c-Myc and the long noncoding RNA (lncRNA) H19, as putative miR-29b upstream regulators, on NG2 expression was assessed.

## Results

### Effect of miR-29b on NG2 expression

**Figure 1:**
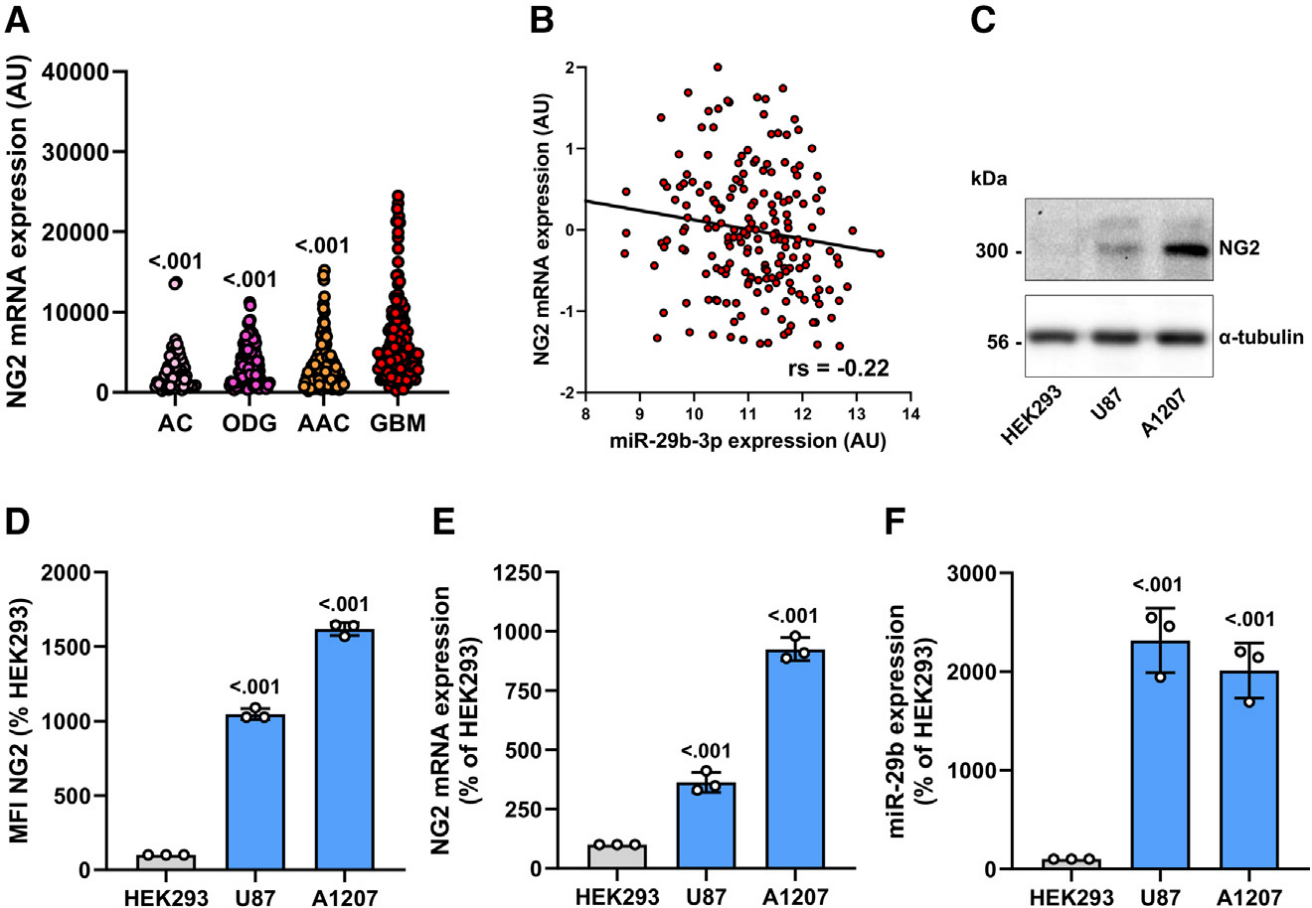
MiR-29b and NG2 expression in GBM and NG2-positive cell lines (A) The relative mRNA expression (TCGA-based data; arbitrary units [AU]) of NG2 was analyzed in human astrocytoma (AC) (n = 67), oligodendroglioma (ODG) (n = 120), anaplastic astrocytoma (AAC) (n = 130), and GBM (n = 152). AC/ODG/AAC vs. GBM. GBM vs. AC: t = 6.53; GBM vs. ODG: t = 6.45; GBM vs. AAC: t = 7.13; F(3, 465) = 26.27. (B) Spearman correlation of NG2 mRNA expression with miR-29b expression (TCGA-based data, n = 198). p = 1.83 × 10^−3^. (C) A1207 and U87 cells were lysed and the expression of NG2 and α-tubulin (as loading control) was analyzed by western blot. Lysed HEK293 cells were used as negative control. (D) A1207 and U87 cells were harvested and the mean fluorescence intensity (MFI) of these NG2-positive cells was assessed by flow cytometry. HEK293 cells were used as negative control and set 100%. Mean ± SD. U87/A1207 vs. HEK293 (n = 3/group). HEK293 vs. U87: t = 35.8; HEK293 vs. A1207: t = 57.35; F(2, 6) = 1678. (E and F) A1207 and U87 cells were harvested, and total RNA was isolated. The relative gene expression of NG2 (E) and miR-29b (F) was examined by qRT-PCR normalized to the corresponding loading control. HEK293 cells were used as negative control and set 100%. Mean ± SD. U87/A1207 vs. HEK293 (n = 3/group). HEK293 vs. U87: t = 8.64; HEK293 vs. A1207: t = 27.04; F(2, 6) = 381.3 (E) and HEK293 vs. U87: t = 10.98; HEK293 vs. A1207: t = 9.48; F(2, 6) = 70.89 (F).

First, we assessed NG2 gene expression in different human gliomas (grade I-IV) based on TCGA data. The results showed that the mRNA expression of NG2 is significantly elevated in GBM when compared with lower-grade gliomas (Figure 1A). Of interest, we further noticed a slightly negative correlation between NG2 and miR-29b in GBM (Figure 1B). To study the effect of miR-29b on NG2 expression, we used the human NG2-positive GBM cell lines A1207 and U87 for the following analyses. These cell lines express miR-29b and NG2, as shown by western blot, flow cytometry, and qRT-PCR (Figures 1C–1F). HEK293 cells, which exhibit a very low NG2 and miR-29b expression, were used as a negative control.

**Figure 2:**
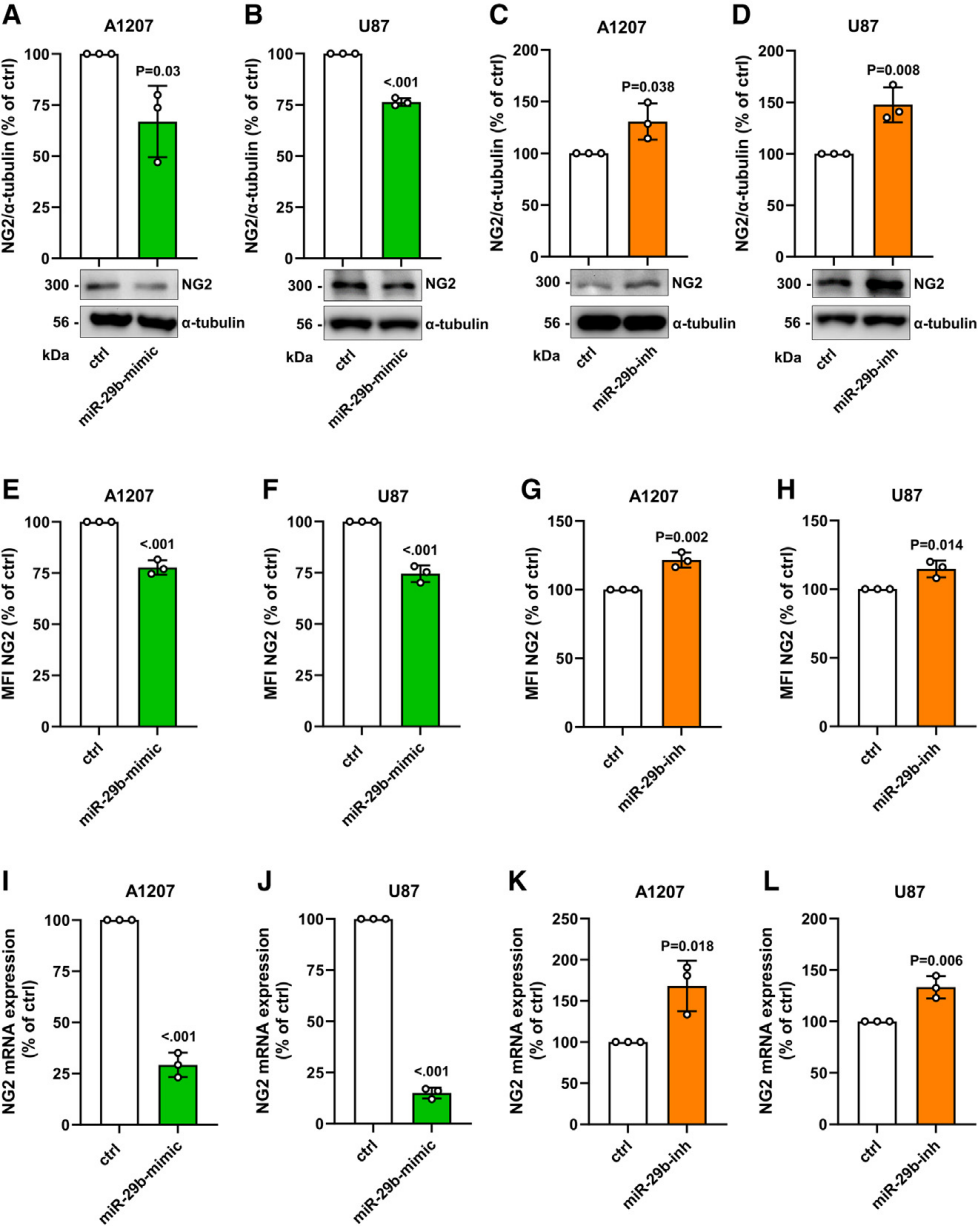
Effect of miR-29b on NG2 expression (A–D) A1207 and U87 cells were transfected with miR-29b-mimic or control (ctrl) (A and B) and miR-29b-inhibitor (miR-29b-inh) or ctrl (C and D) for 48 h. Then, the cells were lysed and the expression of NG2 and α-tubulin (as loading control) was analyzed by western blot (lower panels). Quantitative analyses of NG2/α-tubulin expression (upper panels). Ctrl-transfected cells were set 100%. Mean ± SD. miR-29b-mimic/-inh vs. ctrl (n = 3/group). (E–H) A1207 and U87 cells were transfected with miR-29b-mimic or ctrl (E and F) and with miR-29b-inh or ctrl (G and H) for 48 h. The cells were harvested and the mean fluorescence intensity (MFI) of these NG2-positive cells was assessed by flow cytometry. Ctrl-transfected cells were set 100%. Mean ± SD. miR-29b-mimic/-inh vs. ctrl (n = 3/group). (I–L) A1207 and U87 cells were transfected with miR-29b-mimic or ctrl (I and J) and with miR-29b-inh or ctrl (K and L) for 48 h. The cells were harvested, and total RNA was isolated. The relative gene expression of NG2 was examined by qRT-PCR normalized to GAPDH. NG2 gene expression of ctrl-transfected cells was set 100%. Mean ± SD. miR-29b-mimic/-inh vs. ctrl (n = 3/group).

Next, we analyzed the effect of miR-29b on NG2 expression. By using fluorescence-labeled miR-29b-mimic and scrambled control miR, we detected a high transfection efficiency in A1207 cells (Figure S1). By western blot (Figures 2A–2D) and flow cytometric (Figures 2E–2H) analyses we could demonstrate that overexpression or inhibition of miR-29b significantly reduces or increases NG2 protein levels in A1207 and U87 cells. Additional analyses revealed that miR-29b affects the mRNA levels of the proteoglycan (Figures 2I–2L).

### Posttranscriptional and transcriptional regulation of NG2 by miR-29b

**Figure 3:**
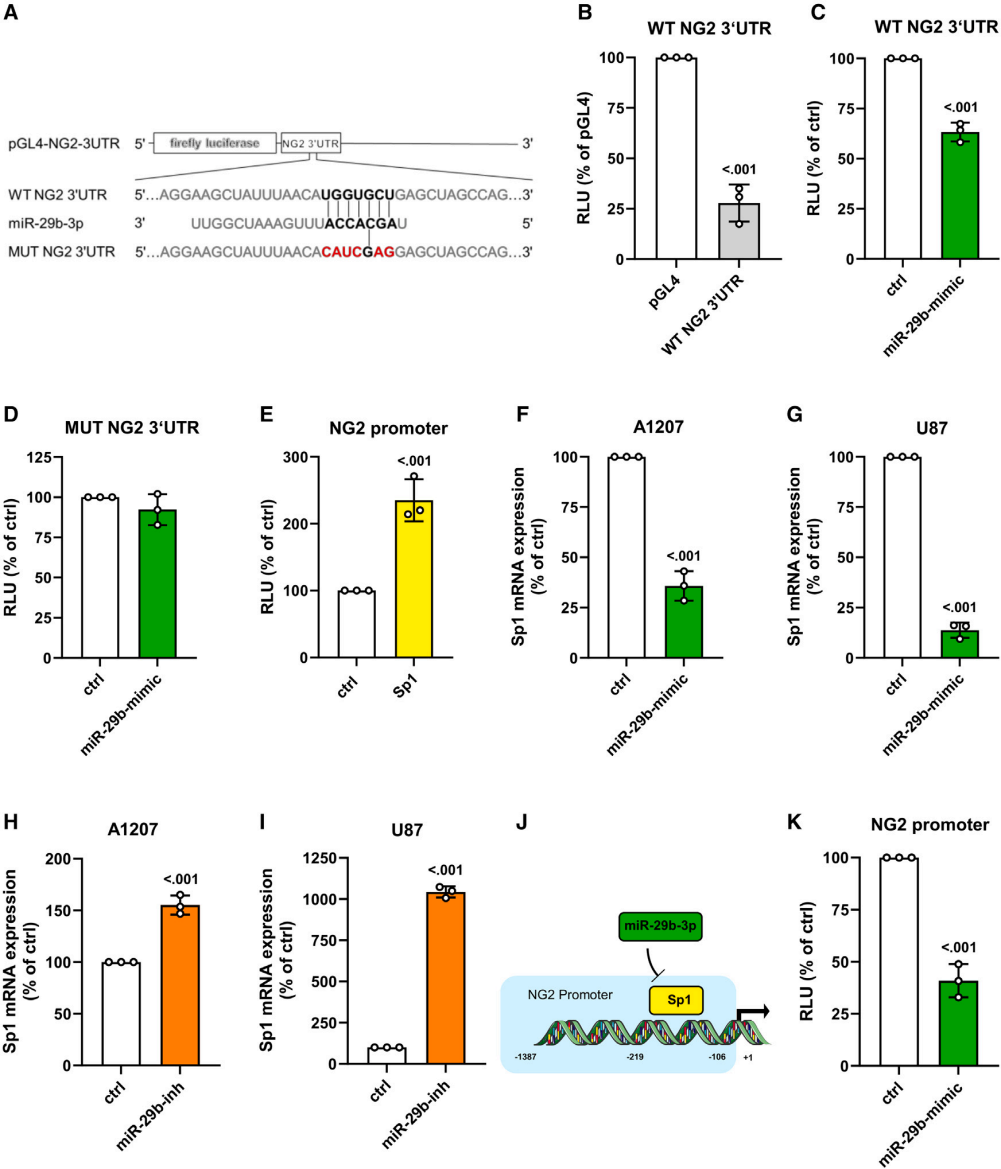
Posttranscriptional and transcriptional regulation of NG2 expression by miR-29b (A) Schematic illustration of miR-29b binding to the wild-type (WT) 3′UTR of NG2 as well as the impaired interaction to the mutated (MUT NG2 3′UTR) binding site. (B) HEK293 cells were transfected with WT NG2 3′UTR or pGL4 empty vector as control for 48 h, lysed, and the activity was detected by a luciferase assay. Relative luciferase units (RLU) of pGL4-transfected cells were set 100%. Mean ± SD. WT NG2 3′UTR vs. pGL4 (n = 3/group). (C and D) HEK293 cells were transfected with WT NG2 3′UTR (C) or MUT NG2 3′UTR (D) and miR-29b-mimic or control (ctrl) for 48 h, lysed, and the activity was detected by a luciferase assay. Relative luciferase units (RLU) of ctrl-transfected cells were set 100%. Mean ± SD. miR-29b-mimic vs. ctrl (n = 3/group). (E) HEK293 cells were co-transfected with the NG2-promoter fragment and Sp1 or empty vector (ctrl) for 48 h, lysed, and the activity was detected by a luciferase assay. Relative luciferase units (RLU) of ctrl-transfected cells were set 100%. Mean ± SD. SP1 vs. ctrl (n = 3/group). (F–I) A1207 and U87 cells were transfected with miR-29b or ctrl (F and G) and with miR-29b-inhibitor (miR-29b-inh) or ctrl (H and I) for 48 h. The cells were harvested, and total RNA was isolated. The relative gene expression of Sp1 was examined by qRT-PCR normalized to GAPDH. Sp1 gene expression of ctrl-transfected cells was set 100%. Mean ± SD. miR-29b-mimic/-inh vs. ctrl (n = 3/group). (J) Schematic illustration of the inhibitory action of miR-29b on Sp1-mediated NG2-promoter activation. (K) HEK293 cells were transfected with the NG2-promoter fragment containing the Sp1 binding site and miR-29b-mimic or ctrl for 48 h, lysed, and the activity was detected by a luciferase assay. Relative luciferase units (RLU) of ctrl-transfected cells were set 100%. Mean ± SD. miR-29b-mimic vs. ctrl (n = 3/group).

*In silico* analyses predicted a binding site of miR-29b in the NG2 3′UTR (Figure 3A).^27^ Therefore, we examined its binding capacity by a luciferase assay. As expected, our results showed that the 3′UTR of NG2 acts posttranscriptionally repressive and overexpression of miR-29b further decreases the luciferase activity (Figures 3B and 3C). Of note, this decrease was abolished by mutation of the binding site (Figures 3A and 3D).

We have recently identified a putative binding site of the transcription factor Sp1 in the NG2 promoter.^19^ In addition, it is known that miR-29b targets Sp1.^28^ Therefore, we next analyzed the effect of miR-29b on NG2 transcription. We found that Sp1 overexpression markedly increases the activity of a 200-base pair (bp) core NG2 promoter element containing the Sp1 site, indicating a direct binding of the transcription factor to the NG2 promoter (Figure 3E). In addition, we detected a lower Sp1 gene expression in miR-29b-mimic transfected NG2-positive GBM cell lines (Figures 3F and 3G). Vice versa, the expression of Sp1 was elevated after transfection of A1207 and U87 cells with miR-29b-inhibitor (miR-29b-inh) (Figures 3H and 3I). Based on these findings, we suggest that miR-29b additionally reduces NG2 gene expression via decreasing Sp1 levels (Figure 3J). In line with this assumption, overexpression of miR-29b repressed NG2 promoter activity (Figure 3K).

### Effect of miR-29b on PDGFRβ and PDGFRα expression

**Figure 4:**
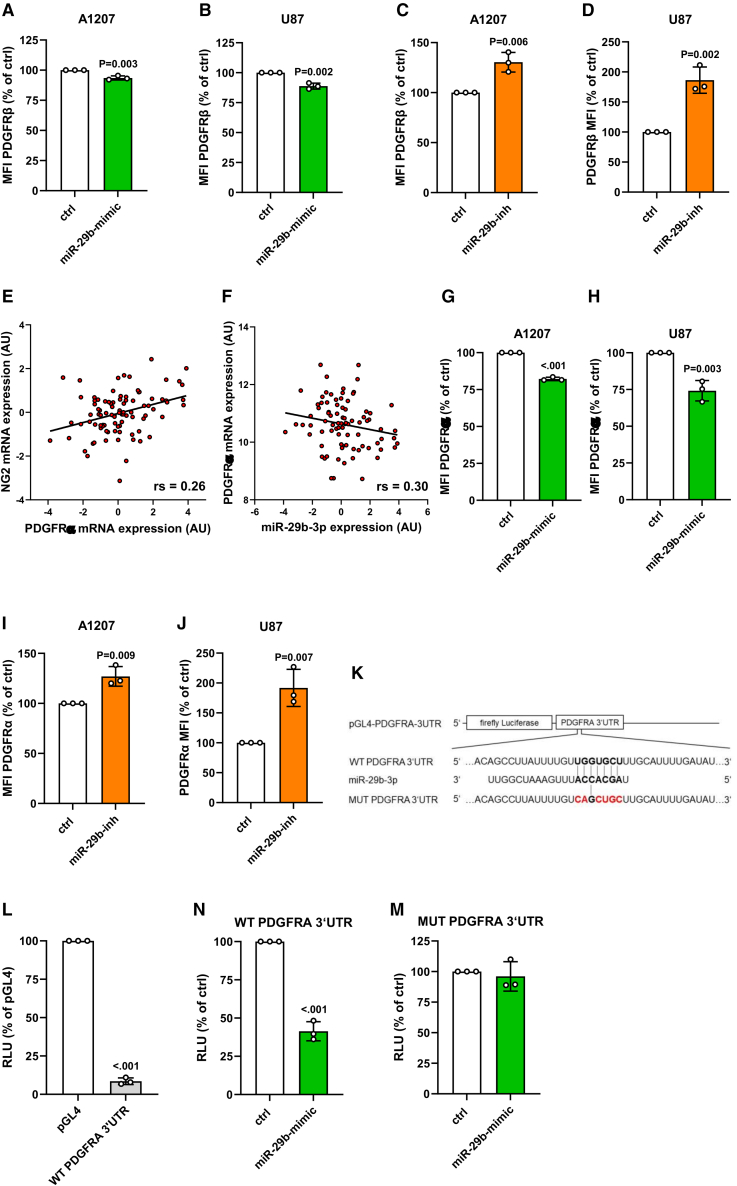
Effect of miR-29b on PDGFRβ and PDGFRα expression (A–D) A1207 and U87 cells were transfected with miR-29b-mimic or control (ctrl) (A and B) and miR-29b-inhibitor (miR-29b-inh) or ctrl (C and D) for 48 h. The cells were harvested and the mean fluorescence intensity (MFI) of these PDGFRβ-positive cells was assessed by flow cytometry. Ctrl-transfected cells were set 100%. Mean ± SD. miR-29b-mimic/-inh vs. ctrl (n = 3/group). (E and F) Spearman correlations of NG2 mRNA expression with PDGFRα mRNA expression (TCGA-based data, n = 91). p = 1.78 × 10^−4^ (E) and of PDGFRα mRNA expression with miR-29b expression (TCGA-based data, n = 84). p = 1.57 × 10^−5^ (F). (G–J) A1207 and U87 cells were transfected with miR-29b or ctrl (G and H) and miR-29b-inh or ctrl (I and J) for 48 h. The cells were harvested, and the MFI of these PDGFRα-positive cells was assessed by flow cytometry. Ctrl-transfected cells were set 100%. Mean ± SD. miR-29b-mimic/-inh vs. ctrl (n = 3/group). (K) Schematic illustration of miR-29b binding to the wild-type (WT) 3′UTR of PDGFRα as well as the impaired interaction to the mutated (MUT PDGFRA 3′UTR) binding site. (L) HEK293 cells were transfected with WT PDGFRA 3′UTR or pGL4 empty vector as control for 24 h, lysed, and the activity was detected by a luciferase assay. Relative luciferase units (RLU) of pGL4-transfected cells were set 100%. Mean ± SD. WT PDGFRA 3′UTR vs. pGL4 (n = 3/group). (N and M) HEK293 cells were transfected with WT PDGFRA 3′UTR (N) or MUT PDGFRA 3′UTR (M) and miR-29b-mimic or ctrl for 48 h, lysed, and the activity was detected by a luciferase assay. Relative luciferase units (RLU) of ctrl-transfected cells were set 100%. Mean ± SD. miR-29b-mimic vs. ctrl (n = 3/group).

NG2 forms clusters with PDGFRα and PDGFRβ, which are involved in PDGF signaling.^11^ PDGFRβ has already been shown as an miR-29b target^29^^,^^30^ and we could verify the regulatory effect of the miRNA on this receptor in NG2-positive GBM cell lines (Figures 4A–4D). However, the effect of miR-29b on PDGFRα expression is still unknown. Our database analyses demonstrated that PDGFRα positively correlates with NG2 expression and negatively with miR-29b expression (Figures 4E and 4F). Additional *in silico* analyses by the TargetScan prediction tool showed that PDGFRα may be targeted by miR-29b (Figure 4K).^27^ Indeed, overexpression of miR-29b significantly reduced whereas the transfection with the inhibitor increased the surface protein level of PDGFRα in A1207 and U87 cells (Figures 4G–4J). We further performed luciferase assays to validate a functional binding site in the 3′UTR of PDGFRA. These analyses revealed that miR-29b decreases PDGFRα protein levels via binding to the 3′UTR (Figures 4L and 4N). As expected, mutation of the binding site abolished the inhibitory effect of the miRNA on 3′UTR of PDGFRA (Figure 4M).

### Effect of miR-29b on NG2-mediated signaling transduction

**Figure 5:**
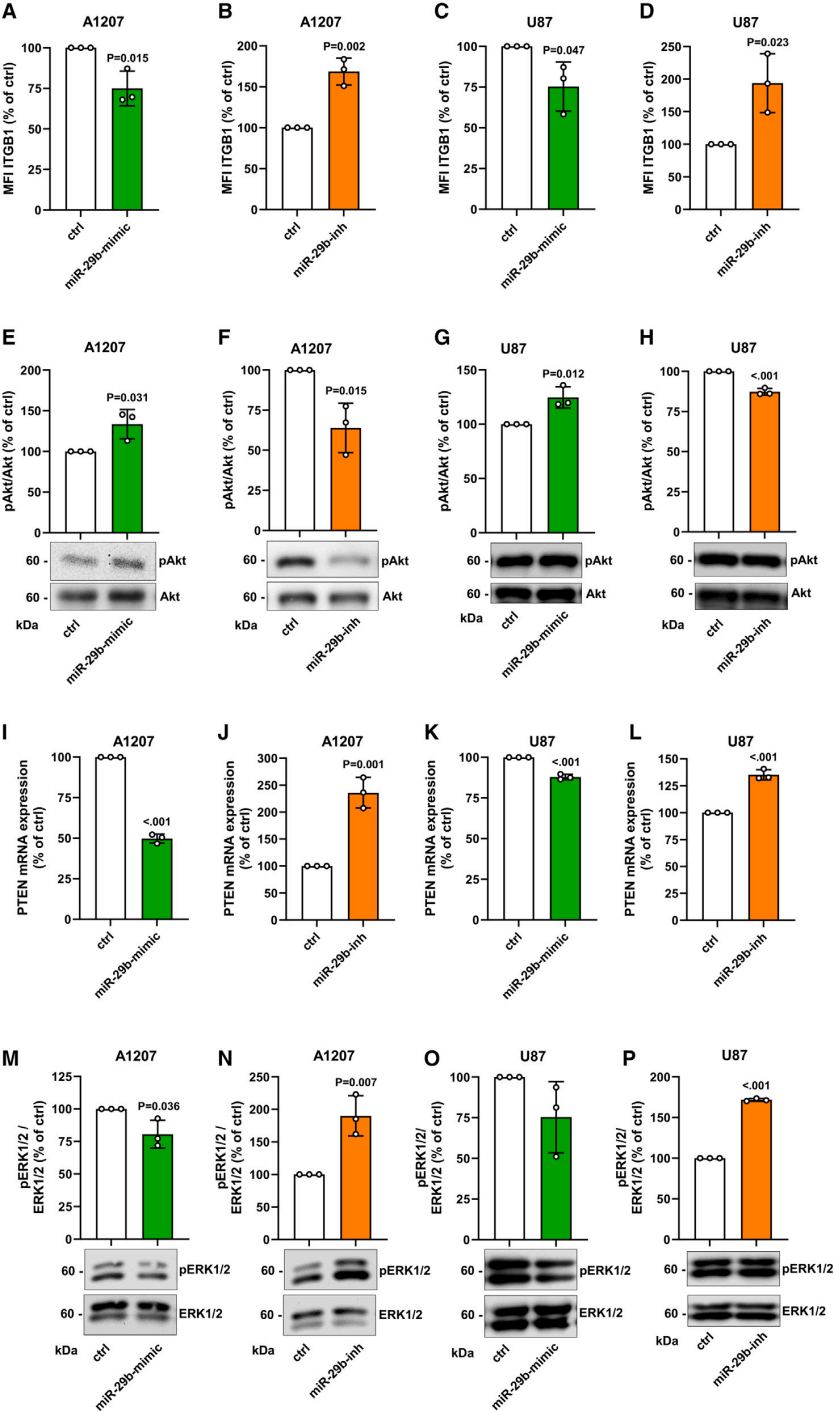
Effect of miR-29b on NG2-mediated intracellular signaling (A–D) A1207 and U87 cells were transfected with miR-29b-mimic or control (ctrl) (A and C) and with miR-29b-inhibitor (miR-29b-inh) or ctrl (B and D) for 48 h. The cells were harvested and the mean fluorescence intensity (MFI) of these ITGB1-positive cells was assessed by flow cytometry. Ctrl-transfected cells were set 100%. Mean ± SD. miR-29b-mimic/-inh vs. ctrl (n = 3/group). (E–H) A1207 and U87 cells were transfected as described in (A–D). Then, the cells were lysed and the expression of pAkt, Akt, and α-tubulin (as loading control) was analyzed by western blot (lower panel). Quantitative analyses of pAkt/Akt expression (upper panel). Ctrl-transfected cells were set 100%. Mean ± SD. miR-29b-mimic/-inh vs. ctrl (n = 3/group). (I–L) A1207 and U87 cells were transfected as described in (A–D). The cells were harvested, and total RNA was isolated. The relative gene expression of PTEN was examined by qRT-PCR normalized to GAPDH. PTEN gene expression of ctrl-transfected cells was set 100%. Mean ± SD. miR-29b-mimic/-inh vs. ctrl (n = 3/group). (M–P) A1207 and U87 cells were transfected as described in (A–D). Then, the cells were lysed, and the expression of ERK1/2, pERK1/2, and α-tubulin (as loading control) was analyzed by western blot (lower panel). Quantitative analyses of pERK1/2/ERK1/2 expression (upper panel). Ctrl-transfected cells were set 100%. Mean ± SD. miR-29b-mimic/-inh vs. ctrl (n = 3/group).

NG2 triggers cell proliferation and migration by activation of FAK and Akt pathways via the interaction with PDGFR and ITGB1.^9^ The latter membrane protein has been shown to be targeted by miR-29b,^29^^,^^31^ which we could confirm by altered surface protein levels as well as mRNA levels in NG2-positive cell lines (Figures 5A–5D and S2A–S2D). Therefore, we expected a significant repression of the above-mentioned pathways after miR-29b-mimic transfection. However, our results showed that miR-29b overexpression promotes the phosphorylation of FAK and Akt, whereas the inhibition of miR-29b diminishes their phosphorylation in A1207 and U87 cells (Figures 5E–5H and S3A–S3D).

In addition, PTEN expression is reduced by miR-29b and, more importantly, this reduction increases Akt signaling.^32^ In line with these results, we herein found a decreased or increased PTEN expression depending on miR-29b expression (Figures 5I–5L and S4A–S4C). On the other hand, NG2 is capable of promoting cell proliferation and migration independently of ITGB1 via upregulation of ERK1/2.^15^ Accordingly, we showed an inverse correlation of miR-29b expression with ERK1/2 phosphorylation in both cell lines (Figures 5M–5P).

### Effect of miR-29b on cell proliferation and migration

**Figure 6:**
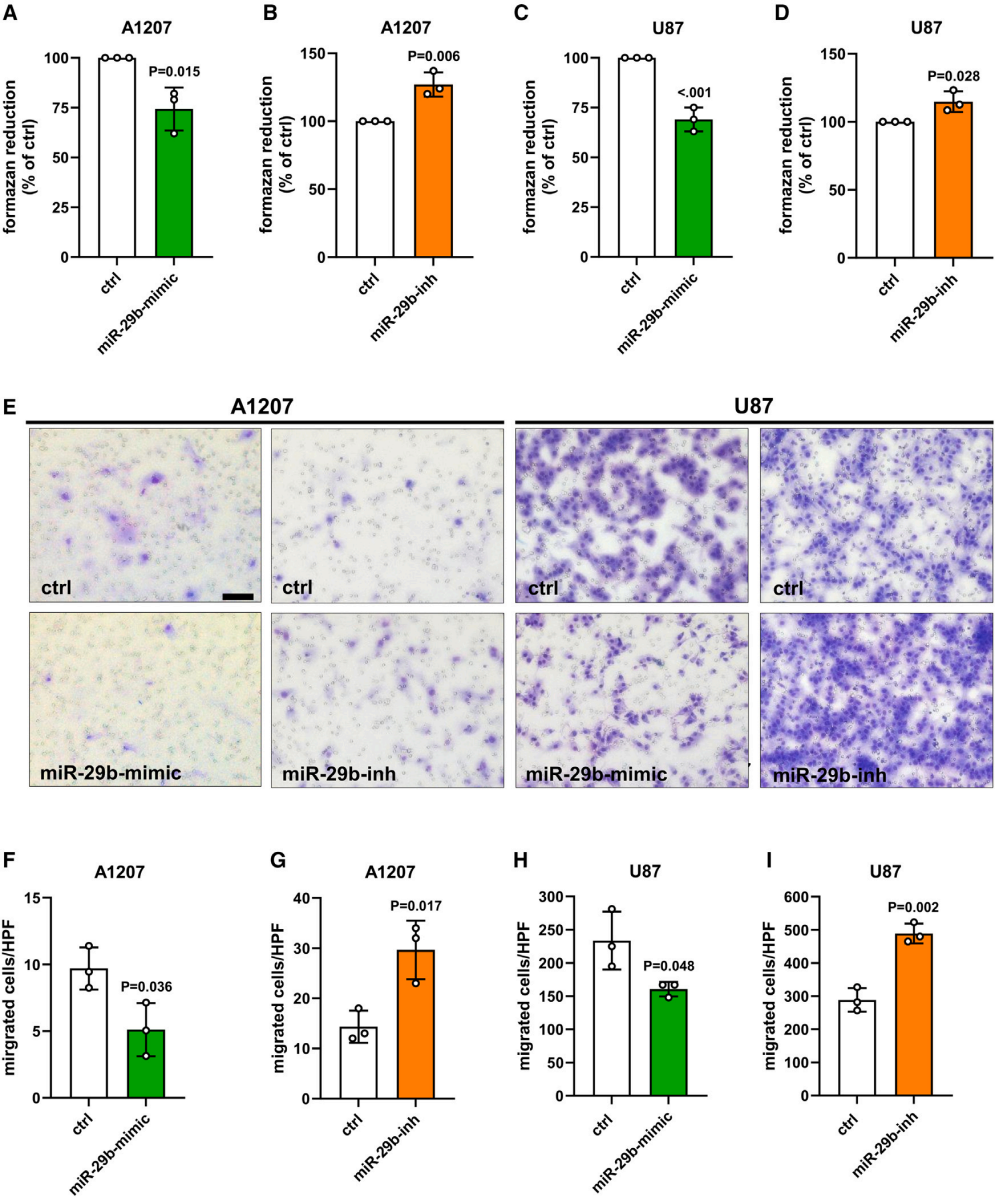
Effect of miR-29b on cell proliferation and migration (A–D) A1207 and U87 cells were transfected with miR-29b-mimic or control (ctrl) (A and C) or with the miR-29b-inhibitor (miR-29b-inh) or ctrl (B and D) for 48 h. Then, the mitochondrial activity was analyzed by a WST-1 assay. Ctrl-transfected cells were set 100%. Mean ± SD. miR-29b-mimic/-inh vs. ctrl (n = 3/group). (E) A1207 and U87 cells were transfected with miR-29b-mimic or ctrl or with the miR-29b-inh or ctrl for 48 h and the migratory capacity was assessed using a transwell migration assay. Bright field images of the migrated cells were taken after 5 h. Scale bar, 50 μm. (F–I) Quantitative analyses of (E). The numbers of migrated cells were counted in 20 high-power fields (HPFs). Data are expressed in % of ctrl-transfected cells. Mean ± SD. miR-29b-mimic/-inh vs. ctrl (n = 3/group).

In the next set of experiments, we assessed the proliferation of NG2-positive GBM cells transfected with miR-29b-mimic or miR-29b-inh. As expected, water-soluble tetrazolium (WST)-1 assays revealed a significantly reduced mitochondrial activity in miR-29b-mimic-transfected A1207 and U87 cells when compared with controls (Figures 6A and 6C). This effect was reversed by miR-29b inhibition (Figures 6B and 6D). Additional scratch and spheroid sprouting assays showed the anti-proliferative and anti-invasive effect of miR-29b (Figures S5A–S5C and S6A–S6C). To selectively study the impact of this miRNA on cell migration, we further performed transwell migration assays. We found that miR-29b suppresses whereas inhibition of the miR-29b promotes migration in both cell lines (Figures 6E–6I). To verify the inhibitory effect of miR-29b on NG2-mediated cell migration in GBM, we additionally overexpressed the miRNA in M059K cells from an NG2-low GBM cell line and analyzed their migratory capacity. We found that miR-29b only slightly reduces the migration of M059K cells, indicating that the effect of miR-29b on cell migration is mainly mediated by reducing NG2 surface protein levels (Figures S7A–S7C).

### Analyses of upstream pathways affecting miR-29b expression

**Figure 7:**
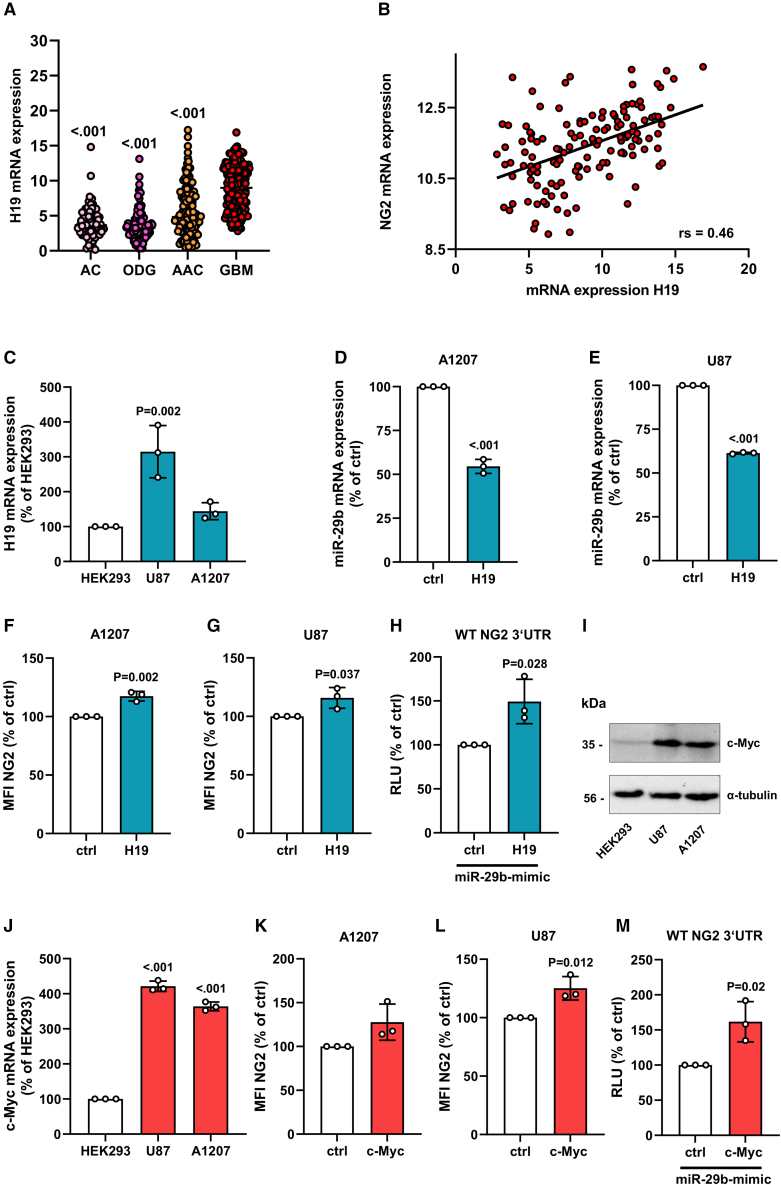
Analyses of pathways affecting miR-29b expression (A) The relative mRNA expression (TCGA-based data; arbitrary units [AU]) of H19 was analyzed in human astrocytoma (AC) (n = 67), oligodendroglioma (ODG) (n = 120), anaplastic astrocytoma (AAC) (n = 130), and GBM (n = 152). AC/ODG/AAC vs. GBM. GBM vs. AC: t = 11.19; GBM vs. ODG: t = 14.38; GBM vs. AAC: t = 9.44; F(3, 478) = 84.97. (B) Spearman correlation of NG2 mRNA expression with H19 expression (TCGA-based data, n = 136). p = 1.24 × 10^−8^. (C) A1207 and U87 cells were harvested, and total RNA was isolated. The relative gene expression of H19 was examined by qRT-PCR normalized to GAPDH. HEK293 cells were used as control and set 100%. Mean ± SD. U87/A1207 vs. HEK293 (n = 3/group). HEK293 vs. U87: t = 5.77; HEK293 vs. A1207: t = 1.18; F(2, 6) = 18.54. (D and E) A1207 (D) and U87 (E) cells were transfected with H19 or empty vector (ctrl) for 48 h. The cells were harvested, and total RNA was isolated. The relative gene expression of miR-29b was examined by qRT-PCR normalized to RNU6. MiR-29b gene expression of ctrl-transfected cells was set 100%. Mean ± SD. H19 vs. ctrl (n = 3/group). (F and G) A1207 (F) and U87 (G) cells were transfected with H19 or empty vector (ctrl) for 48 h. The cells were harvested and the mean fluorescence intensity (MFI) of these NG2-positive cells was assessed by flow cytometry. Ctrl-transfected cells were set 100%. Mean ± SD. H19 vs. ctrl (n = 3/group). (H) HEK293 cells were transfected with WT-NG2-3′UTR and with miR-29b-mimic in combination with H19 or the empty vector (ctrl) for 48 h. The cells were lysed, and the activity was detected by a luciferase assay. Relative luciferase units (RLU) of ctrl-transfected cells were set 100%. Mean ± SD. H19 vs. ctrl (n = 3/group). (I) A1207, U87, and HEK293 cells were lysed and the expression of c-Myc and α-tubulin (as loading control) was analyzed by western blot. (J) A1207 and U87 cells were harvested, and total RNA was isolated. The relative gene expression of c-Myc was examined by qRT-PCR normalized to GAPDH. HEK293 cells were used as control and set 100%. Mean ± SD. U87/A1207 vs. HEK293 (n = 3/group). HEK293 vs. U87: t = 35.32; HEK293 vs. A1207: t = 29.01; F(2, 6) = 709.8. (K–L) A1207 (K) and U87 (L) cells were transfected with c-Myc or empty vector (ctrl) for 48 h. The cells were harvested, and the MFI of these NG2-positive cells was assessed by flow cytometry. Ctrl-transfected cells were set 100%. Mean ± SD. c-Myc vs. ctrl (n = 3/group). (M) HEK293 cells were transfected with WT-NG2-3′UTR and with miR-29b in combination with c-Myc or the empty vector (ctrl) for 48 h. Then, the cells were lysed, and the activity was detected by a luciferase assay. Relative luciferase units (RLUs) of ctrl-transfected cells were set 100%. Mean ± SD. c-Myc vs. ctrl (n = 3/group).

The lncRNA H19 acts as an miR-29b sponge^33^ and, thus, may indirectly affect NG2 expression. We found that H19 is significantly upregulated in GBM when compared with lower-grade gliomas (Figure 7A) and its expression positively correlates with NG2 expression (Figure 7B). The herein used NG2-positive cell lines expressed H19 and overexpression of H19 decreased miR-29b and increased NG2 expression (Figures 7C–7G and S8). To investigate the sponge effect of H19 on miR-29b, we performed a luciferase assay with the 3′UTR of NG2. We could show that overexpression of H19 and miR-29b increases luciferase activity when compared with cells co-transfected with ctrl and miR-29b-mimic (Figure 7H).

Deregulation and/or mutation of c-Myc is present in most GBM cells and considered to be correlated with a poor prognosis of tumor patients.^34^^,^^35^ Moreover, c-Myc upregulates H19 expression (Figure S9).^36^ Therefore, we analyzed whether the expression of NG2 depends on c-Myc activity. We could demonstrate that c-Myc is expressed in NG2-positive GBM cell lines and overexpression of the proto-oncogene elevates NG2 expression (Figures 7I–7L). To verify the stimulatory effect of the c-Myc/H19/miR-29b axis on NG2 expression, we transfected HEK293 cells with the 3′UTR of NG2 and miR-29b in combination with ctrl or c-Myc. By this, we could show that c-Myc promotes NG2 gene expression (Figure 7M).

**Figure 8:**
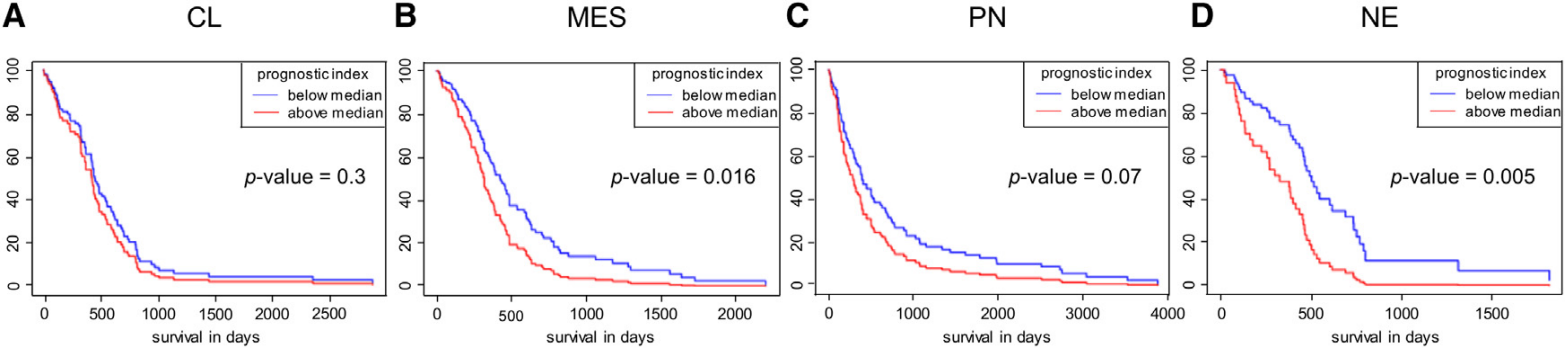
Effect of NG2, PDGFRA, PDGFRB, and ITGB1 expression on patient survival (A–D) Kaplan-Meier survival curves of classical (CL) (n = 104) (A), mesenchymal (MES) (n = 123) (B), proneural (PN) (n = 112) (C), and neural (NE) (n = 55) (D) GBM-subtype patients based on the NG2, PDGFRA, PDGFRB, and ITGB1 expression by constructing a multi-gene prognostic index. Data were retrieved from Glioblastoma Bio Discovery Portal (Glioma-BioDP).^38^^,^^39^

Finally, we correlated the gene expression of NG2, PDGFRA, PDGFRB, and ITGB1, which are all targets of miR-29b, with patient survival, because low miR-29b serum levels are associated with a worse prognosis.^26^ For this purpose, we assessed the prognostic index in the four GBM subtypes: classical (CL), mesenchymal (MES), proneural (PN), and neural (NE) (Figures 8A–8D).^37^ Of interest, our results revealed that low levels of all four genes (NG2, PDGFRA, PDGFRB, and ITGB1) in MES, PN, and NE are associated with an increased patient survival (Figures 8B–8D). This underlines the importance of miR-29b for the survival of GBM patients.

## Discussion

NG2 is strongly associated with a poor prognosis in many cancers by dysregulating central oncogenic pathways, such as FAK, PI3K/Akt, and ERK1/2.^40^ Our TCGA-based data analysis as well as data from other groups^41^^,^^42^ showed that GBM strongly expresses NG2. However, the regulatory mechanisms underlying this elevated NG2 expression in GBM are still unknown. miRNAs play an important regulatory role in tumor development and metastasis. Many miRNAs have been described to be elevated and, thus, to exert an oncogenic function.^43^ For instance, a large-scale analysis of solid tumors revealed an upregulation of the miR-17-92 cluster, which targets the tumor suppressor Rbl2.^44^ On the other hand, miRNAs can also act as tumor suppressors, whereby their expression is dramatically reduced in cancer cells.^45^ MiR-29b exhibits tumor-suppressive activity and, as expected, its expression is decreased in GBM.^24^ Moreover, low levels of exosomal miR-29b are found in serum from GBM patients when compared with the serum from healthy subjects.^26^

Our *in silico* analyses showed that miR-29b may target NG2. In fact, NG2 expression was downregulated after miR-29b overexpression, whereas elevated NG2 levels were detected after inhibition of miR-29b in the herein used NG2-positive GBM cell lines A1207 and U87. Molecular analyses revealed that this is mediated by a direct binding of miR-29b to the 3′UTR of NG2. It has been reported that miR-29b also targets the transcription factor Sp1,^28^ which mediates cell growth and proliferation in GBM.^46^^,^^47^^,^^48^^,^^49^ Of note, we have recently identified an Sp1 binding site in the NG2 promoter.^19^ Hence, we assumed that miR-29b-dependent NG2 expression is further regulated by Sp1. We could show that overexpression of miR-29b reduces Sp1 expression in NG2-positive GBM cell lines as well as its binding to the NG2 promoter. These direct and indirect mechanisms of miR-29b-dependent NG2 expression clearly indicate the pivotal role of miR-29b in the progression of NG2-positive GBM.

NG2 acts as a coreceptor for several membrane proteins, such as PDGFRα and PDGFRβ, which are upregulated in GBM.^37^^,^^50^^,^^51^ PDGFRβ is a well-known target of miR-29b.^29^ However, the effect of this miRNA on PDGFRα expression is still unknown. Our TCGA-database analyses demonstrated that the expression of PDGFRα positively correlates with NG2. Moreover, we found that overexpression of miR-29b decreases PDGFRα protein levels in A1207 and U87 by a direct binding of the 3′UTR. The fact that the heterodimerization of NG2 with PDGFRα is crucial for PDGF-mediated mitogenic signaling^11^ and that miR-29b targets NG2, PDGFRα, and PDGFRβ, reinforces the importance of this miRNA in suppressing NG2-mediated signaling.

PDGFR-mediated signaling transduction results in the activation of the FAK and Akt pathways.^52^^,^^53^ Moreover, Stallcup^9^ reported that silencing of NG2 decreases GBM cell motility, proliferation, and survival by reducing FAK and Akt signaling in an ITGB1-dependent manner. Of note, miR-29b also targets ITGB1.^29^^,^^31^ Based on these findings, miR-29b should diminish the activity of these pathways. However, we detected higher phosphorylation levels of FAK and Akt after miR-29b overexpression. This may be explained by the fact that the miRNA promotes Akt signaling via the downregulation of PTEN.^32^ In line with this observation, we found that miR-29b markedly reduces PTEN expression. NG2 also stimulates cell proliferation via ITGB1-independent mechanisms by potentiation of growth factor signaling. This results in the activation of the ERK1/2 pathway.^15^ ERK1/2 signaling is crucially involved in both cell proliferation and migration.^54^^,^^55^^,^^56^^,^^57^ We herein found that miR-29 is capable of repressing ERK1/2 activity, which, in turn, reduces the proliferation and migration of NG2-positive cell lines. In this context, the group of Cirri^57^ reported that low PDGF concentrations trigger signaling pathways linked to cell motility, whereas high PDGF concentrations activate pathways linked to proliferation. Of note, both processes are partially mediated by ERK1/2.^57^ Therefore, it can be speculated that miR-29b additionally affects ERK1/2 activity via modulating PDGFR signaling. Cattaruzza et al.^58^ identified a functional cooperation of NG2 with FGFR1 and FGFR3. Interestingly, a silico analysis (TargetScan database; data not shown) predicts FGFR1 and FGFR3 as a target of miR-29b. Hence, the direct repression of FGFR1/3-mediated ERK1/2 signaling by miR-29b could further intensify the herein observed anti-proliferative effect.

DNA methylation mediated by DNA methyltransferases (DNMTs) is one of the major mechanisms that govern the epigenetic regulation of the genome. Recently, it has been reported that DNMT expression is associated with TMZ sensitivity in GBM.^59^ Of interest, the miR-29 family is capable of modulating the expression of DNMT.^28^^,^^60^ For instance, Garzon et al.^28^ found that miR-29b not only directly binds to DNMT3A/B but also indirectly suppresses DNMT1 by binding to Sp1. We herein found that miR-29b downregulates the expression of Sp1 in NG2-positive cells. Hence, both direct and indirect epigenetic modifications by miR-29b may contribute to the reduced cell proliferation of NG2-positive cells.

MiR-29b itself is regulated by various mechanisms, including the posttranscriptional interaction with the lncRNA H19.^33^ H19 is abundantly expressed at the fetal stage and repressed in the adult organism with exception of the skeletal muscle system,^61^ where it controls the imprinting of a cluster of conserved genes, such as insulin-like growth factor 2 (IGF2). Studies not only reported an increased expression, but also demonstrated an oncogenic function of H19 in cancer.^62^^,^^63^ Our TCGA-based data showed that H19 is significantly upregulated in GBM and correlates positively with NG2. This indicates that the lncRNA may promote NG2 expression via reducing miR-29b levels. Accordingly, we found that H19 directly binds miR-29b, resulting in an enhanced NG2 expression. Hu et al.^24^ detected high levels of the lncRNA DCST1-AS1 and low levels of miR-29b in GBM tissue when compared with non-tumor tissue. The analysis of the underling mechanism revealed that this is due to a direct binding of DCST1-AS1 to miR-29b. Hence, it is conceivable that additional upregulated lncRNAs in GBM affect miR-29b-mediated NG2 expression.

The Myc proto-oncogene family is causally implicated in most human cancers and the majority of GBMs exhibit elevated c-Myc levels, which are associated with a poor outcome.^34^^,^^35^^,^^64^^,^^65^ The group of Penn^36^ reported that c-Myc significantly induces the expression of H19 in diverse cell types, including GBM. In line with this finding, we could demonstrate a c-Myc-dependent upregulation of NG2 via repression of miR-29b levels. In this context, it should be noted that c-Myc is also capable of directly decreasing miR-29b expression at the transcriptional level to promote tumor aggressiveness.^66^

GBMs are very heterogeneous in their cellular composition and not all GBM cells express NG2. In this context, Al-Mayhani et al.^16^ reported that NG2 expression dramatically differs between GBM samples and GBMs with a high number of NG2-positive cells exhibit an aggressive malignant phenotype.^41^ Hence, this heterogeneity could be crucial for the herein observed weak correlations between miR-29b and NG2, PDGFRα and H19. To exactly determine the expression of miR-29b, PDGFRα, and H19 only in NG2-positive cells, single-cell analyses of GBM samples would be necessary.

Given its central functions in tumorigenesis, NG2 represents a promising target for the treatment of NG2-positive GBM. For instance, several studies demonstrated the successful killing of NG2-positive GBM cells using CSPG4-chimeric-antigen-receptor (CAR)-T cells.^67^^,^^68^^,^^69^ Moreover, we recently found that the pharmacological inhibition of CK2 is a promising mechanism to suppress the proliferation and migration of GBM cells by downregulating NG2 expression.^70^ In the present study, we now demonstrate the great therapeutic potential of miR-29b for the treatment of NG2-positive GBM. In fact, our results indicate that miR-29b does not only inhibit the expression of NG2 but also suppresses the expression of other NG2-interacting proteins, such as PDGFRα, PDGFRβ, and ITGB1. To use this miRNA as a therapeutic molecule in future clinical practice, the development of effective miRNA delivery systems is of major importance. First successful steps toward this direction have been recently taken by the introduction of TargomiRs consisting of bacterially derived minicells with an miRNA and a targeting moiety that are already evaluated in clinical trials (NCT number: 02369198).^71^

The analysis of GBM database (GBM Bio-DP) revealed that low mRNA levels of all four genes (NG2, PDGFRA, PDGFRB, and ITGB1) in MES, PN, and NE subtypes are associated with an increased patient survival. In contrast, this association was not found in the CL subtype. The fact that NG2 interacts with PDGFRα, PDGFRβ, and ITGB1 leads to the assumption that the CL subtype may have low NG2 protein levels and, thus, the signaling transduction mediated via NG2-heterodimer is diminished in this subtype when compared with MES, PN, and NE. To test this hypothesis, additional studies analyzing and correlating the surface protein expression of NG2 as well as PDGFRα, PDGFRβ, and ITGB1 in all GBM subtypes are required.

In summary, we demonstrated that miR-29b directly reduces NG2 expression via binding to the 3′UTR of NG2 as well as indirectly by downregulating the transcription factor Sp1. This could be verified by analyses of TCGA-based data showing a negative correlation between NG2 and miR-29b. We additionally analyzed the effects of miR-29b-mediated NG2 downregulation. Of interest, we could show decreased ERK1/2 activity after miR-29b overexpression, which leads to a markedly reduced tumor cell proliferation and migration. We further identified PDGFRα, a crucial interacting partner of NG2, as a target of miR-29b, which enhances the inhibitory action of miR-29b on GBM progression. Moreover, we identified H19 as a molecular sponge for miR-29b in NG2-positive GBM cells. The expression of H19 is, in turn, induced by the proto-oncogene c-Myc. Taken together, these findings indicate the importance of the c-Myc/H19/miR-29b/NG2 axis for the progression of GBM.

## Materials and methods

### Cell culture

The human GBM cell lines A1207 (SymbioTec GmbH, Saarbrücken, Germany) and U87 (ATCC, Manassas, VA, USA) were cultivated in Roswell Park Memorial Institute medium or Dulbecco’s Modified Eagle Medium supplemented with 10% fetal calf serum (FCS) and penicillin-streptomycin at 37°C and 5% CO_2_ (PAN-Biotech GmbH, Aidenbach, Germany). The cells were passaged at 70% confluence by a split ratio of 1:3 with trypsin-EDTA (Thermo Fisher Scientific, Darmstadt, Germany).

### Antibodies

The anti-NG2 antibody (1:100; sc-166251) was from Santa Cruz Biotechnology (Heidelberg, Germany). The anti-α-tubulin antibody (1:1,000; 66031) was from Proteintech Germany GmbH (St. Leon-Rot, Germany). The antibodies anti-Akt1/2/3 (1:100; 4685), anti-pAkt (1:100; 4060) and anti-c-Myc (1:50; 5605) were from Cell Signaling (Frankfurt am Main, Germany). The anti-ERK1/2 antibody (1:100; ab115799), anti-pERK1/2 antibody (1:100; ab50011) and anti-PTEN antibody (1:50; ab31392) were from Abcam (Cambridge, UK). The peroxidase-labeled anti-rabbit antibody (1:1000; NIF 824) and peroxidase-labeled anti-mouse secondary antibody (1:1,000; NIF 825) were from GE Healthcare (Freiburg, Germany). The PE-labeled anti-chondroitin sulfate proteoglycan 4 antibody (1:30; NG2; 562415), PE-labeled anti-ITGB1 antibody (1:30; 556049), PE-labeled anti-PDGFRα antibody (1:30; 556002), and PE-labeled anti-PDGFRβ antibody (1:30; 55821) were from BD Biosciences (Heidelberg, Germany).

### Reporter constructs

The bioinformatic tool TargetScan predicts a binding site of miR-29b in the 3′UTR of NG2 and PDGFRα.^27^ To verify this *in silico* finding, we inserted the 3′UTR of NG2 and PDGFRα with the miR-29b binding site into pGL4 luciferase reporter vector. PCR products were assembled via Gibson assembly with pGL4 linearized by XbaI restriction (Thermo Fisher Scientific, Darmstadt, Germany). Primer (WT NG2 3′UTR forward 5′- GCGGCAAGATCGCCGTGTAATAATTAGAGCGCCTCCAGTCTAGAAG GCATAAG-3′ and reverse 5′-CCGCCCCGACTCTAGGACAAGGGCCCAGTGCATAGTTAAG-3’; MUT NG2 3′UTR Fragment 1 forward 5′- GCGGCAAGATCGCCGTGTAATAATTAGAGC GCCTCCAGTCTAGAAGGCATAAG-3′ and reverse 5′-CAGTCCTGGCTAGCTCCTCG ATGTGTTAAATAGCTTCC-3′; MUT NG2 3′UTR fragment 2 forward 5′-GGAAGCTATTTAACACATCGAGGAGCTAGCCAGGACTG-3′ and reverse 5′-CCGCC CCGACTCTAGGACAAGGGCCCAGTGCATAGTTAAG-3′; WT PDGFRα 3′UTR forward 5′- GGCGGCAAGATCGCCGTGTAATAATTAAGCTTTGGCGACCCCAATA-3′ and reverse 5′- CGGCCGCCCCGACTCTAGCATGAACAGGGGCATTCGTA-3′; MUT PDGFRA 3′UTR fragment 1 forward 5′- GGCGGCAAGATCGCCGTGTAATAATTAAGCTTTGGCGACCCCAATA-3′ and reverse 5′- CAATATCAAAATGCAAGCAGCTGACAAAATAAGGCTGTAC-3′; MUT PDGFRA 3′UTR fragment 2 forward 5′-GTACAGCCTTATTTTGTCAGCTGCTTGCATTTTGATATTG-3′ and reverse 5′-CGGCCGCCCCGACTCTAGCATGAACAGGGGCATTCGTA-3′) were used at a concentration of 10 μM. The mutation of the miR-29b binding site was generated by overlap extension PCR following Gibson assembly. The vectors were verified by Sanger sequencing.

### Cell transfection

A1207 and U87 cells (2 × 10^4^ per well) were seeded in a 24-well plate. After 24 h, the cells were transfected (HiPerfect, QIAGEN, Hilden, Germany) with 50 nM hsa-miR-29b-3p-5-FAM-labeled (miR-29b-mimic) or with 100 nM hsa-miR-29b-3p inhibitor (miR-29b-inh) (QIAGEN, Hilden, Germany) for 48 h. These concentrations have already been used in other studies.^72^^,^^73^^,^^74^ Cells transfected with scrambled-mimic-5-FAM-labeled or scrambled-inhibitor (QIAGEN, Hilden, Germany) served as controls (ctrl), respectively.

For the overexpression of Sp1,^75^ H19,^76^ and c-Myc, (addgene; Watertown, MA, USA) A1207 and U87 cells (4 × 10^4^ per well) were seeded in a 24-well plate. After 24 h, the cells were transfected (Lipofectamin 3000; Thermo Fisher Scientific, Damstadt, Germany) with 250 ng pEF_Sp1, pcDNA_H19, pcDNA_c-Myc or the corresponding control plasmids (ctrl) for 48 h.

For target validation, HEK293 cells were transfected (Lipofectamin 3000) with pGL4, pGL4_NG2-promoter,^19^ pGL4_WT-NG2-3UTR, pGL4_MUT-NG2-3UTR, pGL4_WT-PDGFRA-3UTR, or pGL4_MUT-PDGFRA-3UTR (250 ng) and co-transfected with the miR-29b-mimic (50 nM) and pEF_Sp1, pcDNA_H19 or pcDNA_c-Myc (250 ng).

### Water-soluble tetrazolium-1 assay

A water-soluble tetrazolium (WST)-1 assay (Roche, Mannheim, Germany) was used to analyze the effect of miR-29b-mimic and miR-29b-inh on the mitochondrial activity of A1207 and U87 cells. Briefly, the cells were seeded in a 96-well culture plate at a density of 2 × 10^3^ cells/well. After 48 h, 10 μL of WST-1 reagent was added into each well and the absorbance was measured at 450 nm in a Tecan Infinite 200 Pro microplate reader (Tecan, Crailsheim, Germany).

### Reporter gene assay

Transfected cells were lysed, and the luciferase activity was measured by Luciferase Reporter Assay (Promega, Walldorf, Germany) using a Tecan Infinite 200 Pro microplate reader (Tecan, Crailsheim, Germany).

### Transwell migration assay

A transwell migration assay was used to analyze the effect of miR-29b-mimic and miR-29b-inh on the migratory capacity of A1207 and U87 cells. For this purpose, 24-well chemotaxis chambers with polycarbonate filters (pore size of 8 μm) (Corning, New York, USA) were preincubated (overnight) in culture medium without any supplements. Thereafter, the medium was removed and 750 μL culture medium supplemented with 5% FCS was added to the bottom well. Subsequently, the cells (2.5 × 10^5^ cells in 200 μL culture medium [0.1% FCS]) were seeded into the upper chamber and incubated for 5 h. Non-migrated cells were removed by cotton swabs and migrated cells were stained with Dade Diff-Quick (Dade Diagnostika, Munich, Germany). The number of migrated cells was counted in 20 high-power fields (HPFs) (BZ-8000; Keyence, Osaka, Japan).

### Western blotting

Transfected cells were harvested and lysed and the protein concentration was measured using a Bradford protein assay (Bio-Rad Laboratories, Feldkirchen, Germany). Bovine serum albumin (BSA) (SERVA Electrophoresis, Heidelberg, Germany) was used as standard. The cell extracts were separated through a 7.5% and 12% SDS-polyacrylamide gel and transferred onto a polyvinylidene difluoride (PVDF) membrane (Bio-Rad Laboratories, Feldkirchen, Germany). After blocking with 5% BSA in tris-buffered saline (TBS) (0.1% Tween 20; 1% BSA) for 1 h, the membrane was incubated with the indicated primary antibodies (dilution 1:100) in TBS (0.1%, Tween 20; 1% BSA) overnight followed by their corresponding secondary antibodies. The protein expression was visualized by enhanced chemiluminescence (ECL) western blotting substrate (Bio-Rad Laboratories, Feldkirchen, Germany) in an ECL ChemoCam Imager (Intas, Göttingen, Germany). The intensity of the measured signals was quantified using ImageJ software and normalized to the loading control (α-tubulin).

### Flow cytometry

Transfected A1207 and U87 cells were washed in phosphate-buffered saline (PBS) and harvested with an enzyme-free dissociation buffer (Thermo Fisher Scientific, Darmstadt, Germany). The cells were incubated with the indicated phycoerythrin (PE)-labeled antibodies for 30 min at room temperature. Then, the cells were washed in PBS and the mean fluorescence intensity (MFI) of 1,000 cells was analyzed by a FACSLyric flow cytometry (BD, Heidelberg, Germany). The non-expressing NG2 cell line HEK293 was used as negative control.

### qRT-PCR

Total RNA was extracted with a QIAzol lysis reagent and transcribed into cDNA by using qScriber (highQu, Kraichtal, Germany). The qRT-PCR analysis was performed by means of ORA SEE qPCR Green ROX L Mix (highQu, Kraichtal, Germany). Forward and reverse primers (NG2 forward 5′-GCAAGCCGATGTGGATTC-3′ and reverse 5′-ATGGCGGATGGTAGGATG-3′; PTEN 5′-GTTTACCGGCAGCATCAAATG-3′ and reverse 5′-CCACTTTAGTGCACAGTTCC-3′; H19 forward 5′-TTCAAAGCCTCCACGACTCT-3′ and reverse 5′-CTGAGACTCAAGGCCGTCTC-3′; Sp1 forward 5′- TGGCTGCCGCTCCCAACTTA -3′ and reverse 5′-ATGATGTTGCCTCCACTTCCTCG-3′; c-Myc forward 5′-CCCACTGCTTACTGGCTTATC-3′ and reverse 5′-CAGCGAGCTCTAGCATTTAGG-3′; glyceraldehyde-3-phosphate dehydrogenase (GAPDH) forward 5′-CCACCCATGGCAAATTCC-3′ and reverse 5′- ACTCCACGACGTACTCAG-3′) were used at a concentration of 500 nM. For miRNA expression analysis, cDNA was prepared by means of miScript II RT Kit (QIAGEN, Hilden, Germany) and analyzed by Quantitect SYBR Green qRT-PCR Kit (QIAGEN, Hilden, Germany). To analyze mature miRNA expression, miScript primer assays for hsa-miR-29b-3p and hsa-U6 small nuclear (RNU6) were used (QIAGEN, Hilden, Germany). Data collection and analyses were performed by the MiniOpticon Real-Time PCR Detection System and the 2^–ΔΔCt^ method using GAPDH and RNU6 as endogenous controls, respectively.

### TCGA data-based analyses

We used two TCGA datasets (Firehose Legacy [Brain Lower-Grade Glioma] and Cell 2013 [Glioblastoma]) from cBioPortal (http://cbioportal.org), an open-access resource for interactive exploration of multidimensional cancer genomics datasets.^77^^,^^78^^,^^79^ RNA Seq V2 RSEM-based data from the TCGA data set (Cell 2013 [Glioblastoma]) were used to analyze the correlations. The multi-gene prognostic index for a group of genes (CSPG4, PDGFRA, PDGFRB, ITGB1) was analyzed by GBM molecular subtype using the National Cancer Institute’s Glioblastoma Bio Discovery Portal software (GBM Bio-DP; https://gbm-biodp.nci.nih.gov).^38^

### Statistical analysis

After testing the data for normal distribution and equal variance, differences between two groups were assessed by an unpaired Student’s t test. To test differences between multiple groups, one-way ANOVA was applied. This was followed by the Bonferroni post hoc test by means of GraphPad Prism (Prism software [version 10.0.3], GraphPad, San Diego, CA, USA). All values are expressed as the mean ± standard deviation (SD). Statistical significance was accepted for p < 0.05. The following statistical values are additionally provided in the figure legends: t = t value and F (Dfn [degrees of freedom numerator], Dfd [degrees of freedom denominator]) = F distribution.

The multi-gene prognostic index (394 samples) was analyzed using a Cox proportional hazards model. Each sample’s prognostic index was determined by averaging individual gene expression from the Cox regression coefficient. The prognostic index was stratified into highest and lowest expression quartiles for each molecular subtype.

## Data and code availability

The data supporting the findings of this study are available from the main text of the article and its supplementary materials.

## Supplemental information

Document S1. Figures S1–S9

Document S2. Article plus supplemental information
