# Supplementary material for: C-Myc/H19/miR-29b axis downregulates nerve/glial (NG)2 expression in glioblastoma multiforme
Source: Mol Ther Nucleic Acids. 2024 Jan 12;35(1):102120. doi: 10.1016/j.omtn.2024.102120 (PMC10839451; doi:10.1016/j.omtn.2024.102120)
Supplement: Document S1. Figures S1–S9 [file mmc1.pdf]

OMTN, Volume *35*

Supplemental information

C-Myc/H19/miR-29b axis downregulates nerve/glial (NG)2 expression in glioblastoma multiforme

Anne S. Boewe, Selina Wrublewsky, Jessica Hoppstädter, Claudia Götz, Alexandra K. Kiemer, Michael D. Menger, Matthias W. Laschke, and Emmanuel Ampofo

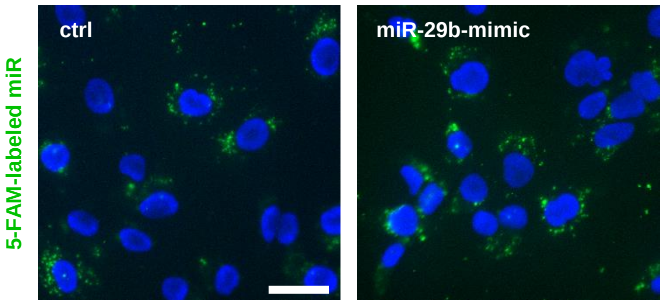

Figure S1. Transfection efficiency. On coverslips seeded A1207 cells were transfected with a 5-FAM-labeled miR-29b or with 5-FAM-labeled ctrl for 48 h. Thereafter, the cells were fixed with PBS containing 3.7% formalin for 10 min, sealed with mounting media and the transfection efficiency was assessed by fluorescence microscopy (BX60; Olympus, Hamburg, Germany). Representative images of 5-FAM-labeled cells (green). Cell nuclei were stained with Hoechst 33342 (blue). Scale bar: 50 μm.

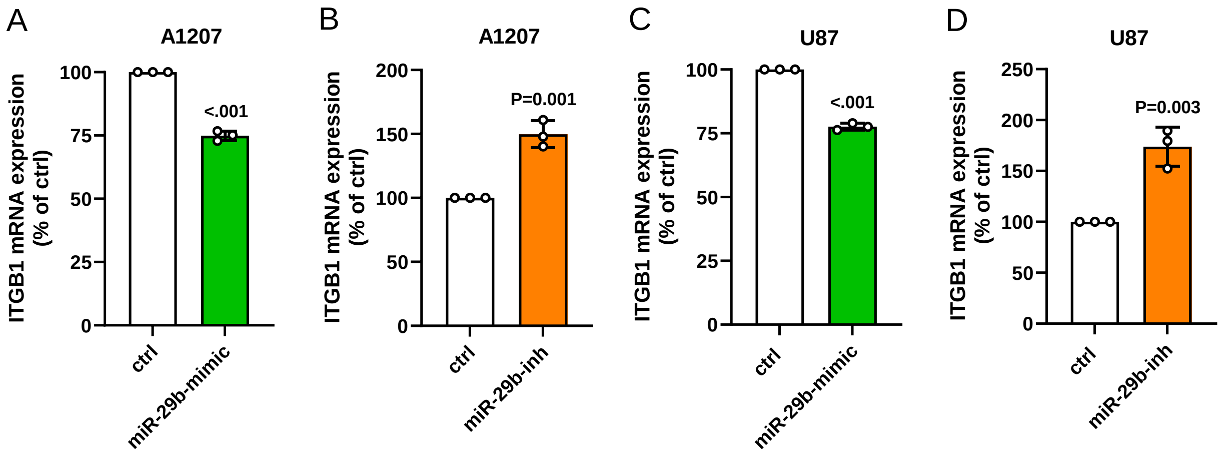

Figure S2. ITGB1 mRNA expression. (**A-D**) A1207 and U87 cells were transfected with miR-29b-mimic or control (ctrl) (A and C) and with miR-29b-inhibitor (inh) or ctrl (B and D) for 48 h. The cells were harvested and total RNA was isolated. The relative gene expression of ITGB1 was examined by qRT-PCR normalized to GAPDH. ITGB1 forward (5'-GGATTCTCCAGAAGGTGGTTTCG-3') and reverse (5'- TGCCACCAAGTTTCCCATCTCC -3') primers were used at a concentration of 500 nM. ITGB1 gene expression of ctrl-transfected cells was set 100%. Mean ± SD. miR-29b-mimic/-inh vs. ctrl (n = 3/group).

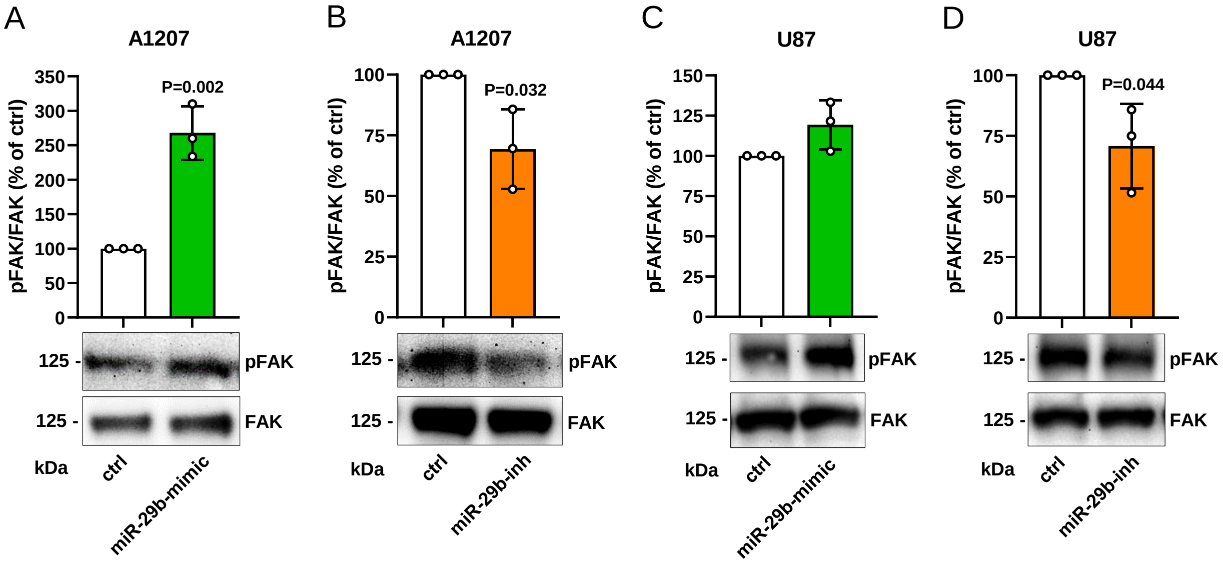

Figure S3. Protein expression of pFAK/FAK. (**A**-**D**) A1207 and U87 cells were transfected with miR-29b-mimic or control (ctrl) (A and C) and with miR-29b-inhibitor (inh) or ctrl (B and D) for 48 h. Then, the cells were lysed and the expression of pFAK and FAK, (anti-FAK (1:100; 3285) and anti-pFAK (1:100; 8556) were from Cell Signaling (Frankfurt am Main, Germany)) was analyzed by western blot. Mean ± SD. miR-29b-mimic/-inh vs. ctrl (n = 3/group).

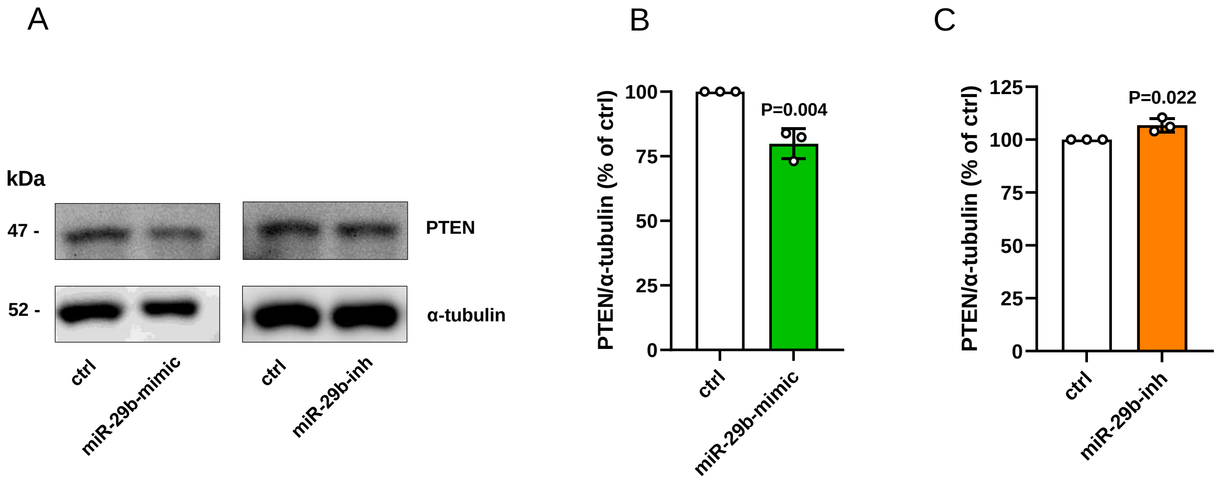

Figure S4. Protein expression of PTEN. (**A**) A1207 cells were transfected with miR-29b-mimic or control (ctrl) and with miR-29b-inhibitor (inh) or ctrl for 48 h. Then, the cells were lysed and the expression of PTEN and α-tubulin (as loading control) was analyzed by western blot. (**B** and **C**) Quantitative analyses of PTEN expression from (A). Ctrl-transfected cells were set 100%. Mean ± SD. miR-29b-mimic/-inh vs. ctrl (n = 3/group).

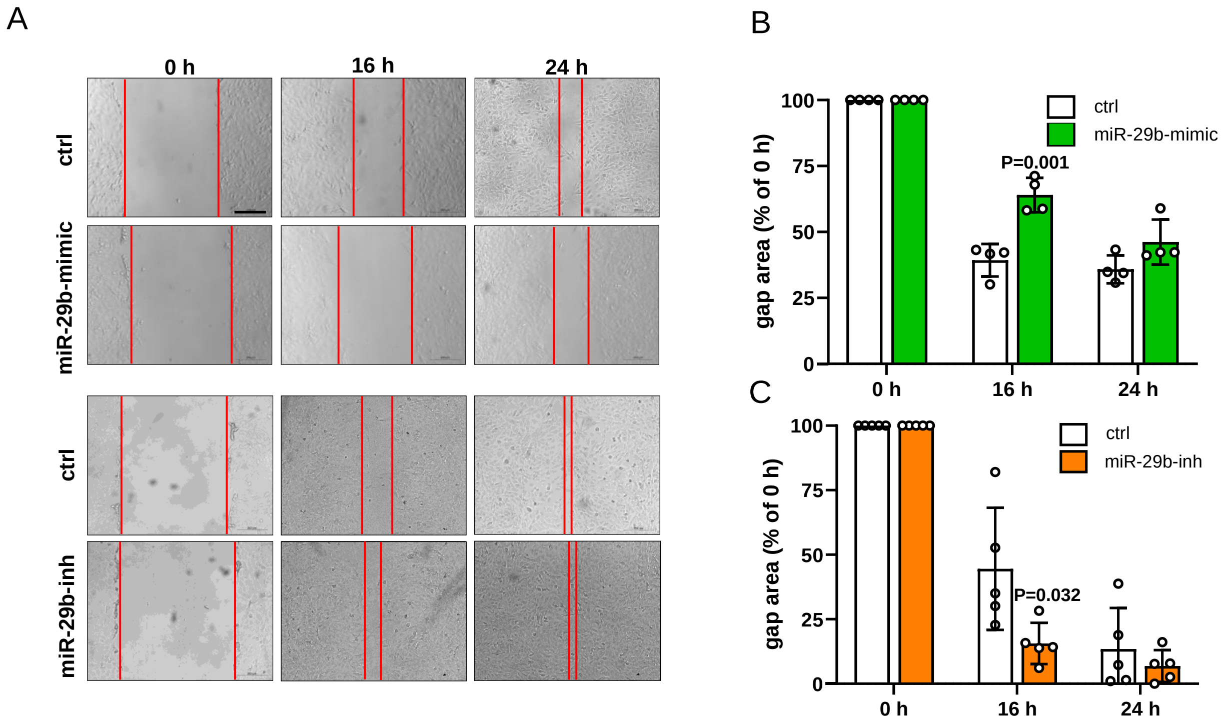

Figure S5. Scratch assay. (**A**) The migratory capacity of miR-29b-mimic- and miR-29b-inhibitor (inh)-transfected A1207 cells was assessed by a scratch assay. For this purpose, the cell monolayer was scratched with a pipette tip and rinsed twice with PBS to remove non-adherent cells. Phase-contrast light microscopic images were taken immediately (0 h), 16 h and 24 h after scratching. Scale bar: 200 µm. (**B** and **C**) The gap area was measured by means of ImageJ software after 0 h, 16 h and 24 h (U.S. National Institutes of Health (NIH), Bethesda, Maryland, USA). The gap area at 0 h was set 100%. Mean ± SD. miR-29b-mimic/-inh vs. ctrl (n = 4-5/group).

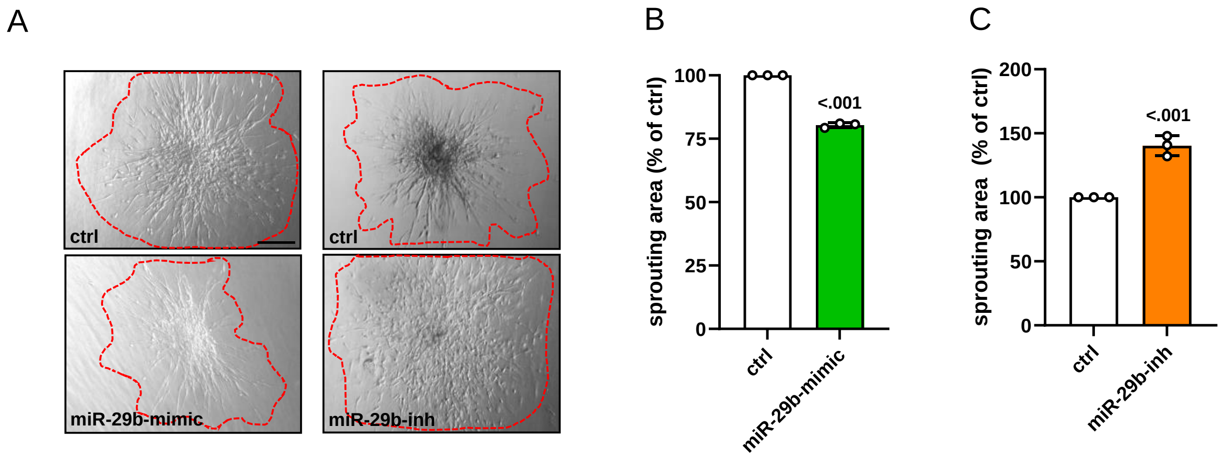

Figure S6. Spheroid sprouting assay. (**A**) MiR-29b-mimic- and miR-29b-inhibitor (inh)-transfected A1207 cells were harvested and 500 cells per well were seeded into 96-well plates coated with 40 µL 1% agarose to form spheroids. After 24 h, 40 spheroids were collected, mixed in a collagen solution and transferred into a well of a 24-well plate. After 45 min, RPMI medium was added to the wells and the spheroids were incubated for 48 h. The sprouting capacity (borders marked by broken red lines) was visualized by bright field microscopy. Scale bar: 200 µm. (**B** and **C**) The sprouting area was measured by the ImageJ software (NIH, Bethesda, Maryland, USA). Data are expressed in % of control (ctrl)-transfected spheroids. Mean ± SD. miR-29b-mimic/-inh vs. ctrl (n = 3/group).

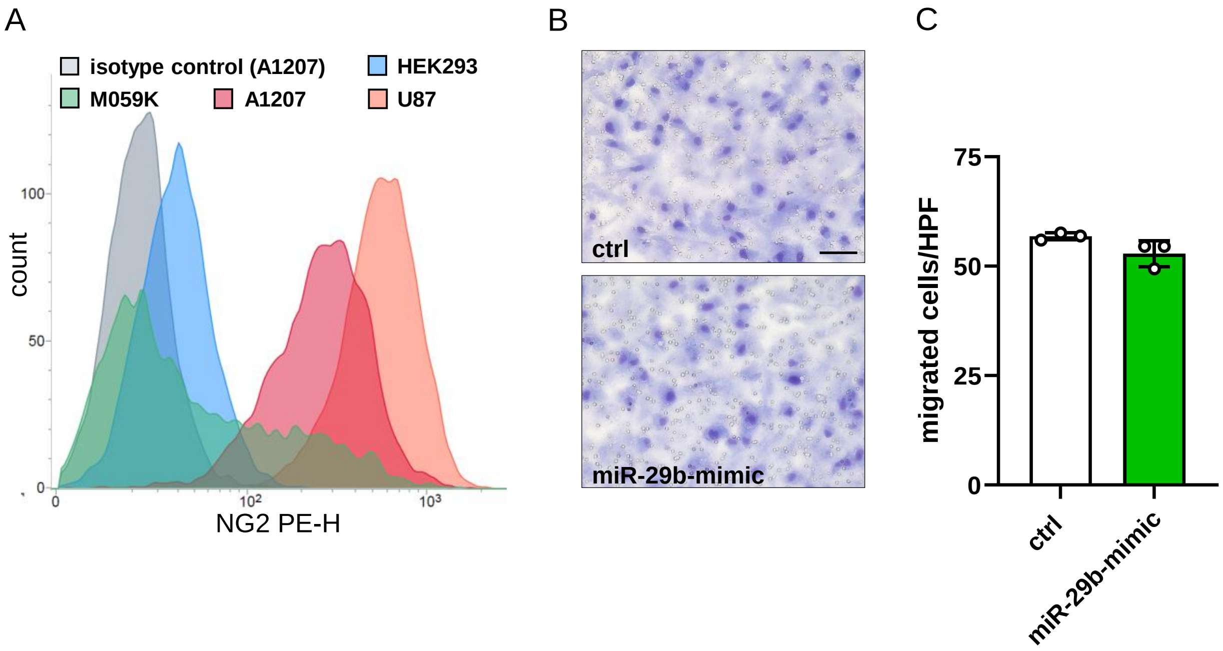

Figure S7. Transwell migration assay. (**A**) Expression of NG2 in HEK293 (blue), M059K (green), A1207 (red) and U87 (orange). A1207 labeled with the isotype PE antibody (grey) served as negative control. Representative histograms were generated from flow cytometric analyses of 3000 cells. (**B**) MiR-29b-mimic-transfected M059K and control (ctrl)-transfected cells were seeded in 24-well chemotaxis chambers to study their migratory capacity. After 5 h incubation, the number of migrated cells was counted in 20 high-power fields (HPF). Scale bar: 50 µm. (**C**) Quantitative analyses of migrated cells per HPF. Mean ± SD (n = 3/group).

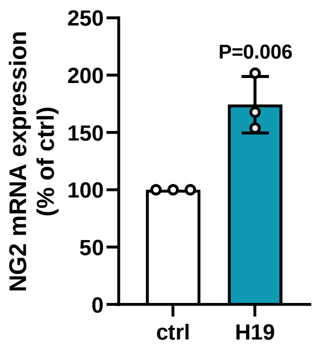

Figure S8. NG2 mRNA expression. A1207 cells were transfected with H19 or empty vector (control (ctrl)) for 48 h. The cells were harvested and total RNA was isolated. The relative gene expression of NG2 was examined by qRT-PCR normalized to GAPDH. NG2 gene expression of ctrl-transfected cells was set 100%. Mean ± SD. H19 vs. ctrl (n = 3/group).

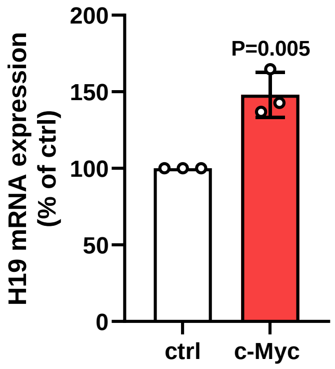

Figure S9. H19 mRNA expression. A1207 cells were transfected with c-Myc or empty vector (control (ctrl)) for 48 h. The cells were harvested and total RNA was isolated. The relative gene expression of H19 was examined by qRT-PCR normalized to GAPDH. H19 gene expression of ctrl-transfected cells was set 100%. Mean ± SD. c-Myc vs. ctrl (n = 3/group).
